# Gradient boosting for yield prediction of elite maize hybrid ZhengDan 958

**DOI:** 10.1371/journal.pone.0315493

**Published:** 2024-12-17

**Authors:** Oumnia Ennaji, Sfia Baha, Leonardus Vergutz, Achraf El Allali

**Affiliations:** 1 College of Computing, University Mohammed VI Polytechnic, Benguerir, Morocco; 2 College of Agriculture and Environmental Sciences, University Mohammed VI Polytechnic, Benguerir, Morocco; ICAR Indian Agricultural Statistics Research Institute, INDIA

## Abstract

Understanding accurate methods for predicting yields in complex agricultural systems is critical for effective nutrient management and crop growth. Machine learning has proven to be an important tool in this context. Numerous studies have investigated its potential for predicting yields under different conditions. Among these algorithms, Random Forest (RF) has gained prominence due to its ability to manage large data sets with high dimensions, as well as its ability to uncover complicated non-linear relationships and interactions between variables. RF is particularly suitable for scenarios with categorical variables and missing data. Given the complex web of management practices and their nonlinear effects on yield prediction, it is important to investigate new machine learning algorithms. In this context, our study focused on the evaluation of gradient boosting methods, particularly Extreme Gradient Boosting (XGB) and Gradient Boosting Regressor (GBR), as potential candidates for yield estimation of the maize hybrid Zhengdan 958. Our aim was not only to evaluate and compare these algorithms with existing approaches, but also to comprehensively analyze the resulting model uncertainties. Our approach includes comparing multiple machine learning algorithms, developing and selecting suitable features, fine-tuning the models by training and adjusting the hyperparameters, and visualizing the results. Using a recent dataset of over 1700 maize yield data pairs, our evaluation included a spectrum of algorithms. Our results show robust prediction accuracy for all algorithms. In particular, the predictions of XGB (*RMSE* = 0.37, *R*2 = 0.87 and *MAE* = 0.26) and GBR(*RMSE* = 0.39, *R*2 = 0.86 and *MAE* = 0.27), emphasized the central role of weather characteristics and confirmed the high dependence of crop yield prediction on environmental attributes. Utilizing the capabilities of gradient boosting for yield prediction holds immense potential and is consistent with the promise of this method to serve as a catalyst for further investigation in this evolving field

## Introduction

Improving management decisions within crop production systems holds great importance in narrowing yield gaps and safeguarding food security. However, conventional approaches to agriculture have struggled to keep pace with the escalating demands of more intricate systems, particularly those involving small, scattered farms. These challenges are exacerbated by the misalignment of management recommendations with production systems and environmental variables, as observed in the literature [1]. One of the factors leading to low profitability and inefficient use of inputs is low knowledge of limiting factors. Historically, understanding region-specific best practices has relied on controlled experiments within small plots, followed by dissemination through extension services. This methodology has yielded benefits but falls short in addressing contemporary challenges, including climate change, management, and labor supply.

The anticipation of crop yields stands as an important element in sustainable cultivation and optimal utilization of natural resources. However, the intricacies involved in yield prediction render it an extremely complex task influenced by many factors [2, 3]. This complexity prevents both researchers and farmers from accurately forecasting yields and the subsequent economic gains over both short and extended time frames. Prior to the advent of machine learning, conventional statistical models, field surveys, and simulation models were used in diverse combinations to explain and predict crop-yield dynamics [4].

In recent times, machine learning techniques have emerged as potentially effective tools for probing the factors influencing crop yields and for yield prediction itself [1, 5–7]. The integration of these methods has the potential to enhance precision in nutrient management, encourage sustainable cultivation, and fortify food security. Machine learning-based tools can foster the preservation of biodiversity while concurrently augmenting crop yields across various cropping systems, soil compositions, and climatic conditions [8, 9]. Notably, each algorithm offers unique insights into the complexity of these challenges. Maize, being a model species, has been scrutinized extensively for yield and fertilization aspects in diverse experimental setups across global locations. Recent studies have showcased the integration of machine learning in various hybrid configurations.

**Fig 1:**
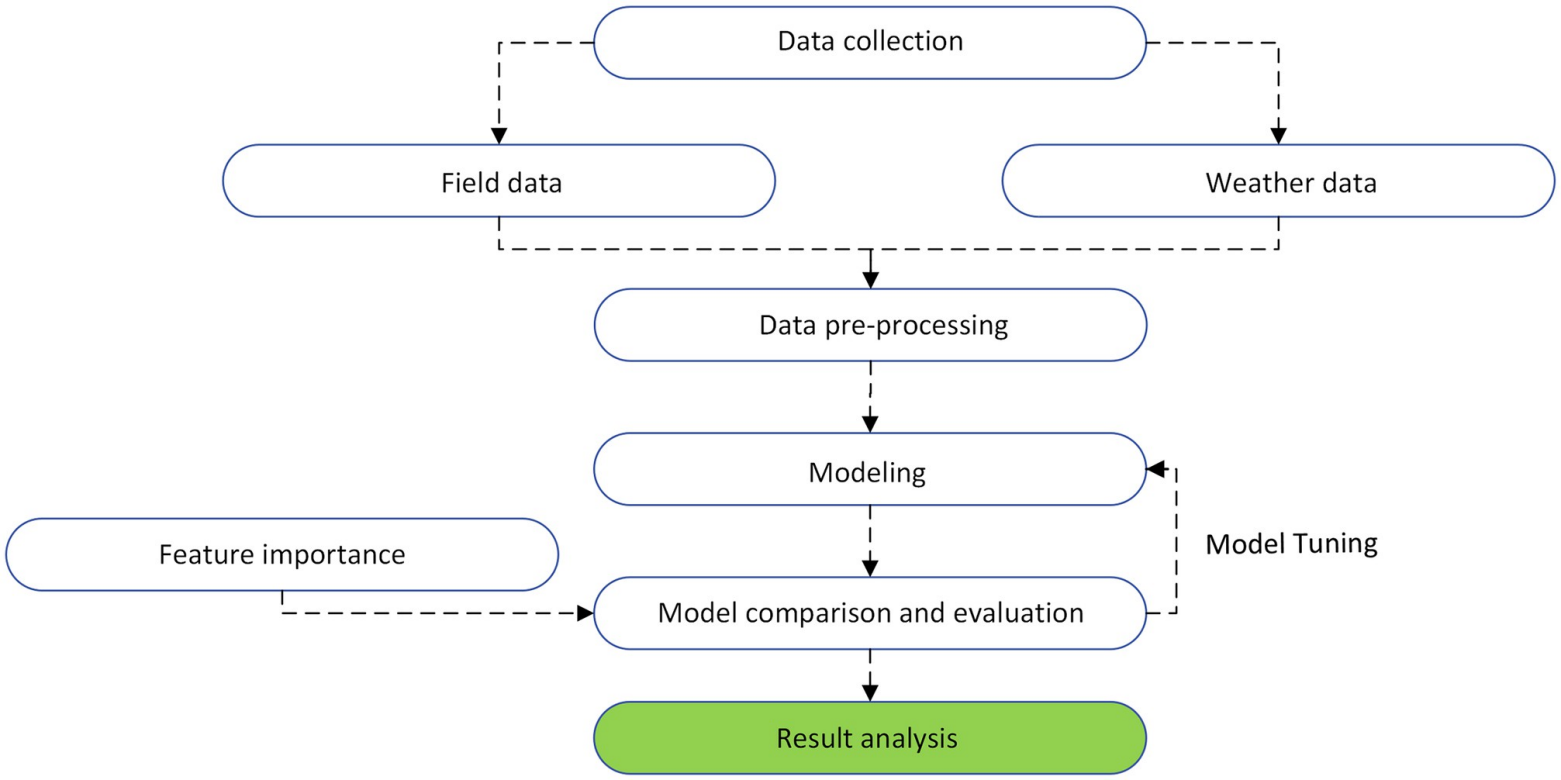
General workflow of the machine learning approach used in the current study.

The research by [10] established a structured approach to distinguish between the spatial and temporal aspects of crop yield, enabling the assessment of different data types in predicting yields and grasping their connection to underlying mechanisms. By employing multiple linear regression (MLR) and random forest (RF), the authors highlighted the importance of within-field location in modeling corn and soybean yields in Nebraska. Other researchers, such as [11], coupled machine learning with spatiotemporal soil fertility data to predict corn yields. In addition, [12] leveraged satellite-based climate and vegetation indices to forecast maize yields for smallholder farmers, effectively demonstrating a scalable yield estimation method even in data-scarce scenarios. Further contributions include a comprehensive evaluation of corn yield prediction, wherein six machine learning algorithms were tested alongside environmental variables from satellite observations [13]. [14] explored diverse machine learning models, including XGBoost, Gradient Boosting Machine, Random Forest, Decision Tree, Adaptive Boosting, and Neural Network, to forecast corn hybrid yields. XGBoost and GBM showed good predictive results, with XGBoost standing out due to its advanced regularization techniques. Such studies have helped shape the advancement of yield prediction modeling. In the context of maize (Zea mays L.), a staple food with important economic significance, yield is influenced by an array of variables and environmental dynamics [15–18]. This study focuses on assessing the predictive performance of gradient51 boosting regressors for corn yield prediction, including XGBoost and GBR [19, 20]. The investigation included a comparative analysis, involving other machine learning algorithms used to predict yields from high-dimensional data, in order to compare them with GB-based algorithms. The primary goal of this study is to explore the responsiveness of yields to diverse environmental variables, evaluate the contribution of weather variables to seasonal crop yield predictions, and discern disparities in the performance of distinct machine learning algorithms, particularly in the case of the ZhengDan 958 hybrid and GB-based machine learning algorithms. A schematic overview of the methodology is presented in Fig 1.

For this particular study, the focus centers on summer maize, which has a critical role in the food security of China, the second largest global producer and importer of maize [21]. Comprehending the intricate interplay between climate and corn production within the Chinese context is critical and scientists have undertaken numerous investigations on the subject [22, 23]. In maize breeding, inbred lines diverge into two primary categories: the Stiff Stalk Heterosis Group (SS group) and the Non-Stif Stalk Heterosis Group (NSS group), based on the degree of grain yield heterosis. Typically, intragroup crossings exhibit lower grain yield heterosis than intergroup crosses. The study’s focal point is the hybrid Zhengdan 958, a prominent maize hybrid recurrently cultivated in China. Its parent inbred lines stem from distinct heterosis groups: Zheng 58 pertains to the PA subgroup of modern U.S. hybrid-derived germplasm (part of the stiff stalk heterosis group—SS group), while Chang 7–2 associates with the Tsipingtou (TSPT) heterosis group, a subset of the non-stiff stalk heterosis group (NSS group) [24]. Noteworthy attributes of Zhengdan 958 involve yield, planting density, and stress resilience. However, few studies evaluating yield parameters were found in the literature [25–27]. Our study contributes to these efforts by showcasing the important features linked to yield in this important maize hybrid.

## Materials and methods

### Study site and dataset

The study area focuses on China. Maize one of the most important cereal, has increased rapidly in China from 23.1 Mha (106 hectares) in 2000 to 42.1 Mha in 2018. The average yield has also risen from 4.60 tons per hectare to 6.10 tons per hectare in the same period. In the North China Plain (NCP), smallholders grow spring maize (Zea mays L.) together with winter wheat and achieve average maize yields of 5.39 Mg ha-1 [28]. The summer maize variety ZhengDan958, the most commonly grown variety in China, was used as test material. No irrigation was applied during the entire growing season except for sowing. The study area includes 1700 data points in NCP. In crop modeling, a multitude of agronomic principles are meticulously considered to produce precise features related to their impact and significance on crop growth and the evolution of yield. In our pursuit, we harmonized various datasets to construct an appropriate groundwork for our machine-learning workflow. Our initial step entailed preparing the dataset curated by [29], with a specific focus on isolating Zhengdan 958 from other cultivars during the June to September timeframe of the years 2005 to 2010. We identified meteorological indicators that influence yield production [6]. Weather data from diverse sources were homogenized and adjusted to the same temporal and spatial resolution. The result is a comprehensive dataset, encompassing a spectrum of parameters such as temperature (inclusive of minimum, maximum, and average temperatures at a height of 2 meters), humidity and precipitation, atmospheric pressure (surface pressure), and soil characteristics alongside NPK applications. Table 1 summarizes the final features used in to build the models.

**Table 1:** Description of the features used to build the yield prediction models for Zhengdan 958.

Dataset	Variables
**Coordinates**	Latitude (N)
	Longitude (E)
**Soil Features**	SOM(g kg-1)
	Olsen-P(mg kg-1)
	Ava-K(mg kg-1)
	N input (kg ha-1)
	K2O input (kg ha-1)
	P205 input (kg ha-1)
**Weather Features**	Temperature at 2 Meters Range (°C)
	Temperature at 2 Meters Maximum (°C)
	Temperature at 2 Meters Minimum (°C)
	Temperature at 2 Meters (°C)
	Relative Humidity at 2 Meters (%)
	Precipitation (mm/day)
	Surface Pressure (kPa)
**Yield**	Yield (kg -1)

### Methodology

A number of different predictive models were trained to predict yields, encompassing eXtreme Gradient Boost (XGB), Gradient Boosting (GBR), Random Forest (RF), Artificial Neural Network (ANN), Support Vector Regression (SVR), Linear Regression (LR), Decision Trees (DT), and K-Nearest Neighbors (KNN). The eXtreme Gradient Boost (XGB) algorithm employs a gradient boosting technique fine-tuned for decision and regression trees. The Gradient Boosting Regressors (GBRs) utilize an iterative approach to refine response variable estimates by adapting new models. Central to this methodology is the development of base learning models exhibiting high correlation with the negative gradient of the ensemble’s associated loss function. In contrast, Random Forest (RF) constructs multiple regression trees using feature subsets and bootstrap samples, combining outcomes for robust predictions.

Multilayer Perceptron (MLP), an artificial neural network, consists of input, hidden and output layers. MLPs use backpropagation for training and can learn complex, non-linear relationships between input features and target output values. With multiple hidden layers and non-linear activation functions such as ReLU or Sigmoid, MLPs can capture interactions in the data that simpler models may miss. In this study, we use an MLP for our regression task of yield forecasting. The MLP maps input variables such as weather, soil and management data to continuous yield values.

The Support Vector Regression (SVR) algorithm attempts to locate the hyperplane (or line, in simple linear regression) that maximizes the gap between predicted and actual values. Linear Regression (LR) learns from data by minimizing loss typically quantified as RMSE or MSE through algorithms like gradient descent [30]. Decision Trees (DT), a widely employed algorithm, addresses both regression and classification tasks, with nodes representing data attributes and branches symbolizing decisions or rules based on these attributes. The final result is represented by the leaf nodes. K-Nearest Neighbors (KNN), on the other hand, is a versatile machine-learning algorithm handling both regression and classification. This algorithm compares the similarity of features between a new data point and the already labeled data points in its vicinity.

To confirm the non-linearity of the data, we performed the Teraesvirta test for neural networks [31]. The test statistic was 669.109 with a p-value of 10^−16^, indicating a strong rejection of the null hypothesis of linearity and confirming the presence of non-linear relationships in the dataset. This result strongly supports the appropriateness of employing non-linear models, such as gradient boosting in our analysis. The test was conducted using a random seed of 42 to ensure reproducibility.

**Fig 2:**
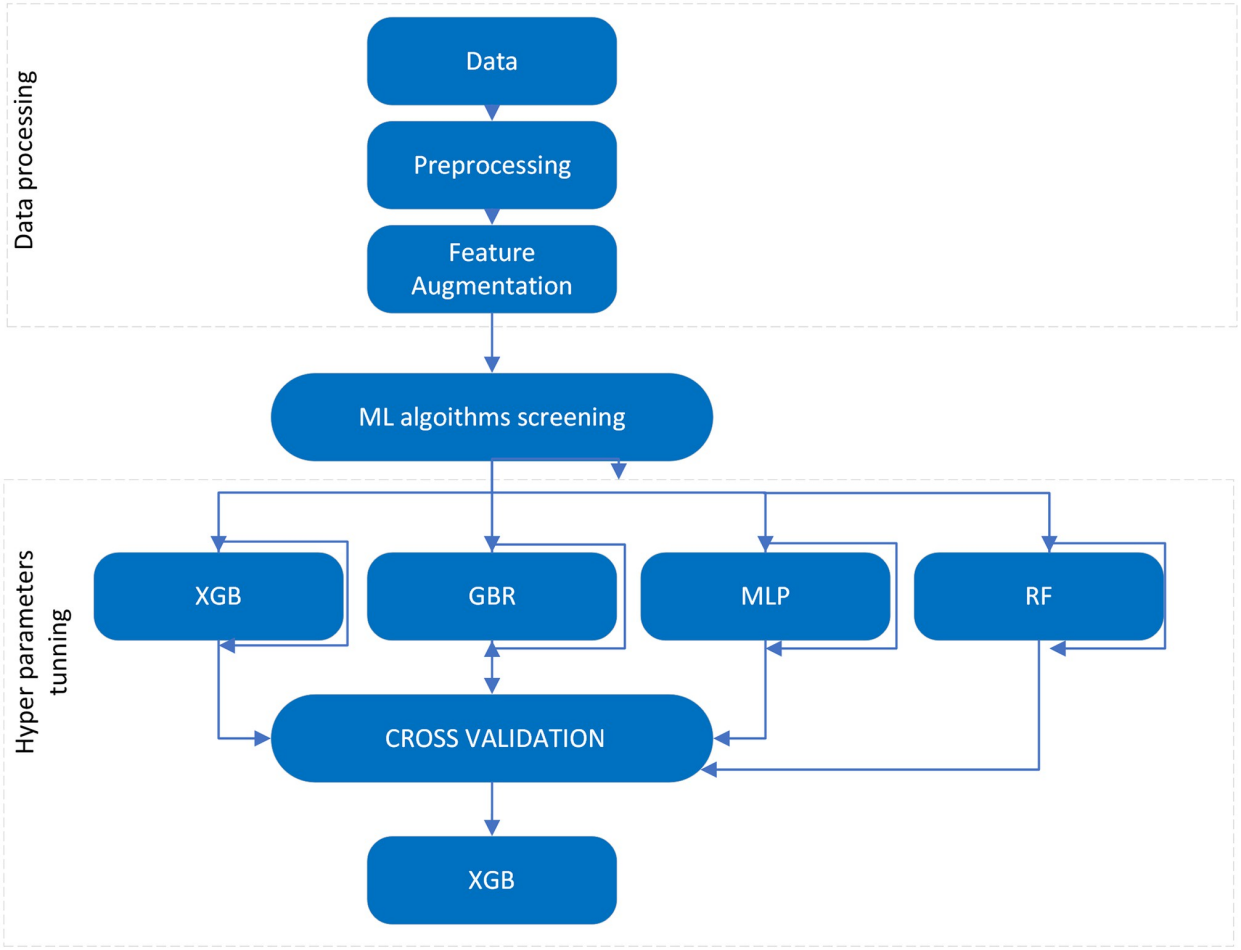
Workflow diagram showing the steps from data processing, model training, and testing.

For each algorithm, a grid search across the hyperparameter space was employed to find the best-performing parameters using 5-fold cross-validation. Consequently, the most efficient hyperparameters were used to build and evaluate each models. Fig 2 shows the overall framework followed in our study.

The performance of the models was assessed using three metrics: root mean squared error (RMSE), mean absolute error (MAE), and coefficient of determination (*R*^2^) [5]. These metrics are outlined in Eqs 1, 2 and 3:

$$\begin{array}{c} RMSE=\sqrt{\frac{1}{n}\sum_{i=1}^{n}{\left(yi-\hat{yi}\right)}^{2}} \end{array}$$ (1)

$$\begin{array}{c} R^{2}=1-\frac{\sum_{i=1}^{n}{\left(yi-\hat{yi}\right)}^{2}}{\sum_{i=1}^{n}{\left(yi-\bar{yi}\right)}^{2}} \end{array}$$ (2)

$$\begin{array}{c} MAE=\frac{1}{n}\sum_{i=1}^{n}|yi-\hat{yi}| \end{array}$$ (3)

Here, *yi* is the actual measured yield, $\hat{yi}$ is the predicted yield, and $\bar{yi}$ represents the mean of the actual measured yield.

## Results

### Prediction performance

**Fig 3:**
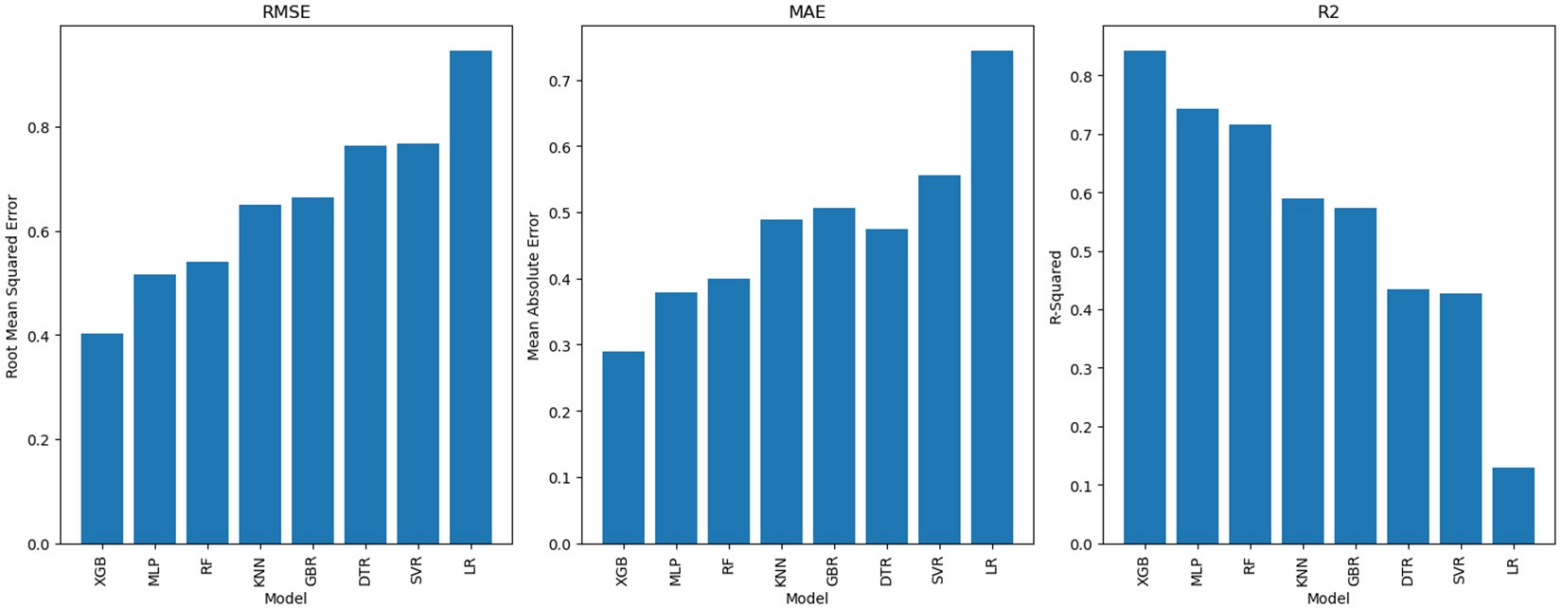
Comparison of the performance of ML algorithms in predicting maize yield using 5-fold CV. The performance of the models was assessed using RMSE, *R*^2^ and MAE.

One of the objectives of this study is to compare the performance of XGBoost (XGB) and Gradient Boosting Regressor (GBR) with that of Random Forest (RF), which are commonly used for yield prediction. In addition to these models, we evaluated MLP, KNN, DT, SVR and LR for a comprehensive comparison of predictive performance. The results in Table 2 and Fig 3, show that XGBoost has the highest prediction accuracy with an *R*^2^ of 0.84, an RMSE (0.40) and an MAE (0.29). GBR, which was developed similarly to XGBoost, achieved an *R*^2^ of 0.57, with an RMSE of 0.66, MAE of 0.51. RF, showed a strong performance with an *R*^2^ of 0.72, an RMSE of 0.54, MAE of 0.40. Models such as MLP also performed well, with an *R*^2^ of 0.74 and an RMSE of 0.52, but models such as KNN, DT, SVR and LR showed comparatively lower performance, with *R*^2^ values between 0.13 and 0.59 and higher RMSE values. The cross-validation results show that XGBoost consistently delivered the best generalization performance with the lowest MSE across all models. This highlights the robustness of XGBoost across different data splits, in contrast to models such as GBR and RF, which had higher cross-validation errors, indicating greater variability in performance on unseen data. While XGBoost significantly outperformed both GBR and RF in terms of prediction accuracy and stability, both GBR and RF performed competitively. Detailed results including training and testing performance measures can be found in the supplementary material (S4 Table). Overall, the study shows that XGBoost provides the best overall performance, with RF and GBR also showing strong results with slightly higher cross-validation errors than XGBoost. Fig 3 illustrates the RMSE, MAE and *R*^2^ using a 5-fold cross-validation.

**Table 2:** RMSE, MAE and *R*^2^ of the models using 5-fold CV.

Model	RMSE	MAE	R2
XGB	0.41	0.29	0.84
MLP	0.53	0.38	0.72
RF	0.55	0.40	0.71
KNN	0.65	0.49	0.59
GBR	0.66	0.51	0.57
DTR	0.74	0.47	0.47
SVR	0.77	0.56	0.43
LR	0.95	0.74	0.13

XGB, MLP, RF, and GBR were selected for further optimization using hyperparameter tuning. Table 3 highlights that both XGB and GBR outperformed RF in terms of RMSE, *R*^2^, and MAE values. Specifically, XGB and GBR achieved RMSE values of 0.37 and 0.39, *R*^2^ values of 0.87 and 0.86, and MAE values of 0.26 and 0.27, respectively. In contrast, the results of hyperparameter tuning for RF were relatively lower, with an RMSE of 0.71, an *R*^2^ of 0.51, and an MAE of 0.56. MLP, while performing better than RF, showed weaker performance compared to both XGB and GBR, with an RMSE of 0.51, an *R*^2^ of 0.75, and an MAE of 0.38. These results demonstrate that XGB and GBR, after hyperparameter tuning, offer superior predictive performance in comparison to both MLP and RF, particularly in terms of minimizing RMSE and maximizing *R*^2^. To confirm the best-performing model, we conducted the Diebold-Mariano test [32], which revealed that XGBoost significantly outperforms all other models in terms of forecasting accuracy. The results show a statistically significant improvement in performance, confirming XGBoost as the most accurate model among those tested. Full test results are available in the supplementary material (S5 Table).

**Table 3:** The predictive performance of XGB, GBR, MLP, and RF after hyper-parameters tuning (the highest accuracy was highlighted in bold).

Algorithm	RMSE	R2	MAE
**XGB**	**0.37**	**0.87**	**0.26**
GBR	0.39	0.86	0.27
MLP	0.51	0.75	0.38
RF	0.71	0.51	0.56

**Fig 4:**
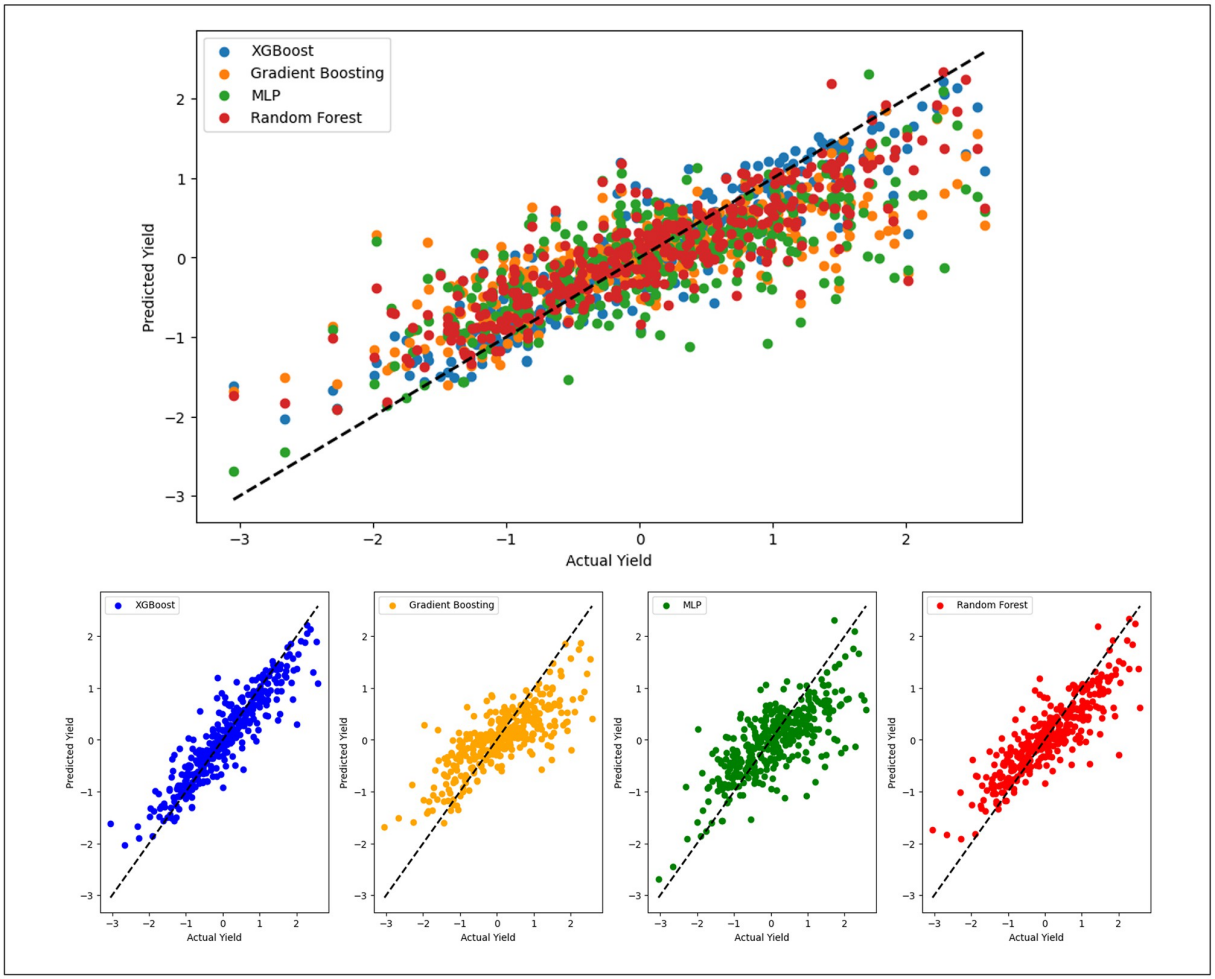
Comparison between predicted and actual crop yield of each model: XGB, GBR, MLP and RF.

To illustrate the comparative results, we generated plots depicting the predicted versus actual yield for the selected algorithms. Fig 4 illustrates the correlation between the projected and actual yield. Evidently, a positive-sloped linear regression line is shown within the plots. While some values exhibit marginal deviations from the line, these anomalies may be attributed to bias effects. However, the visual analysis consistently reaffirms the performance of XGBOOST,GBR, MLP and RF, substantiating their effectiveness in yielding the most accurate results.

**Fig 5:**
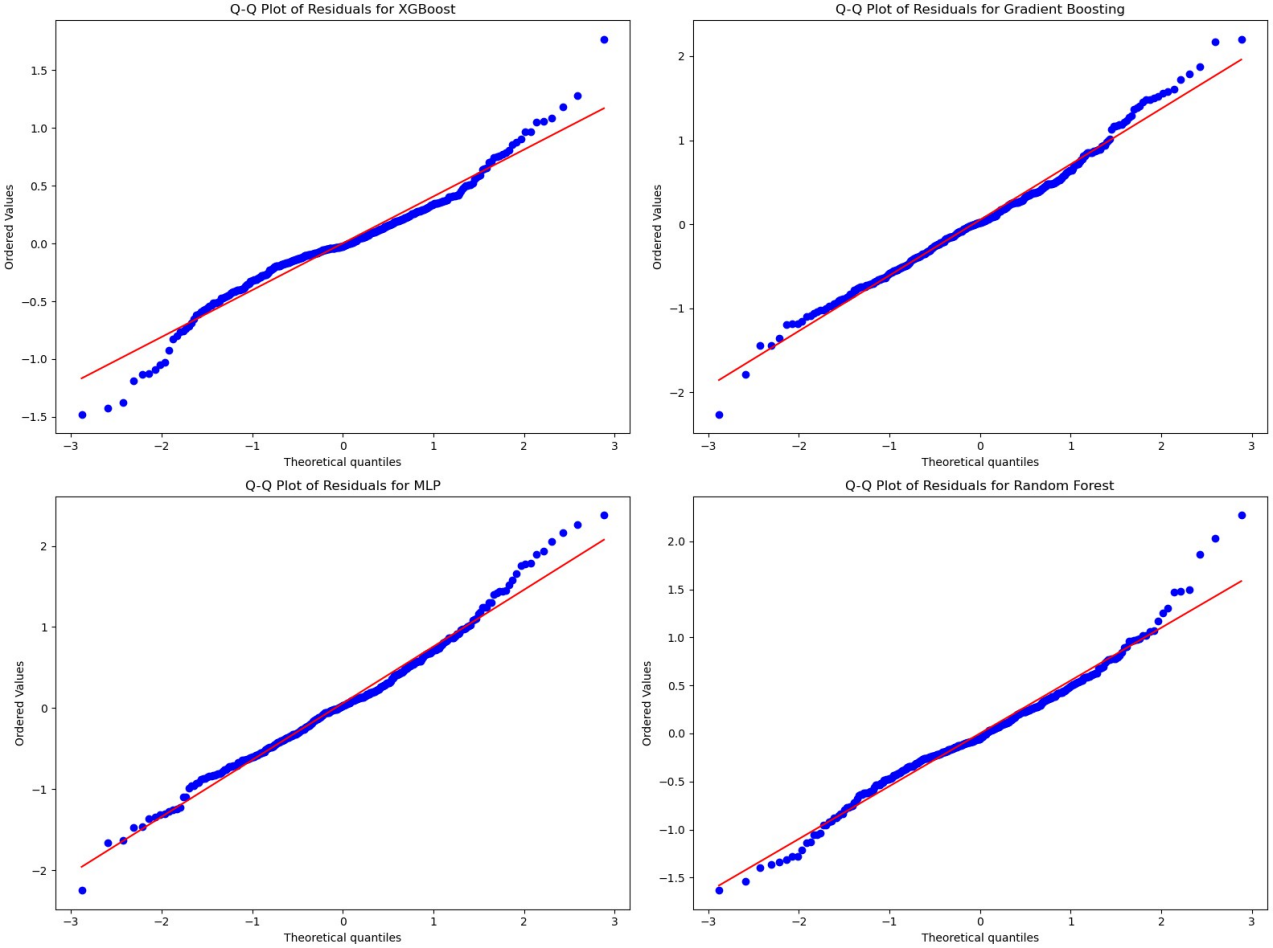
Residual Quantile-Quantile (QQ) plot for each model: XGB, GBR, MLP, and RF (QQ plot compares the observed residuals against the standardized normal distribution.

We performed normality tests on the residuals for each of the models, as presented in Fig 5. The results indicated that the residuals for both the Gradient Boosting Regressor (GBR) and eXtreme Gradient Boosting (XGB) models showed slight deviations from normality. This outcome is typical for non-linear models, where such deviations are common. However, these deviations do not substantially impact the predictive performance of the models, as GBR and XGB do not rely heavily on normality assumptions. Detailed test results are available in the supplementary material S1 and S2 Figs.

### Feature importance

**Fig 6:**
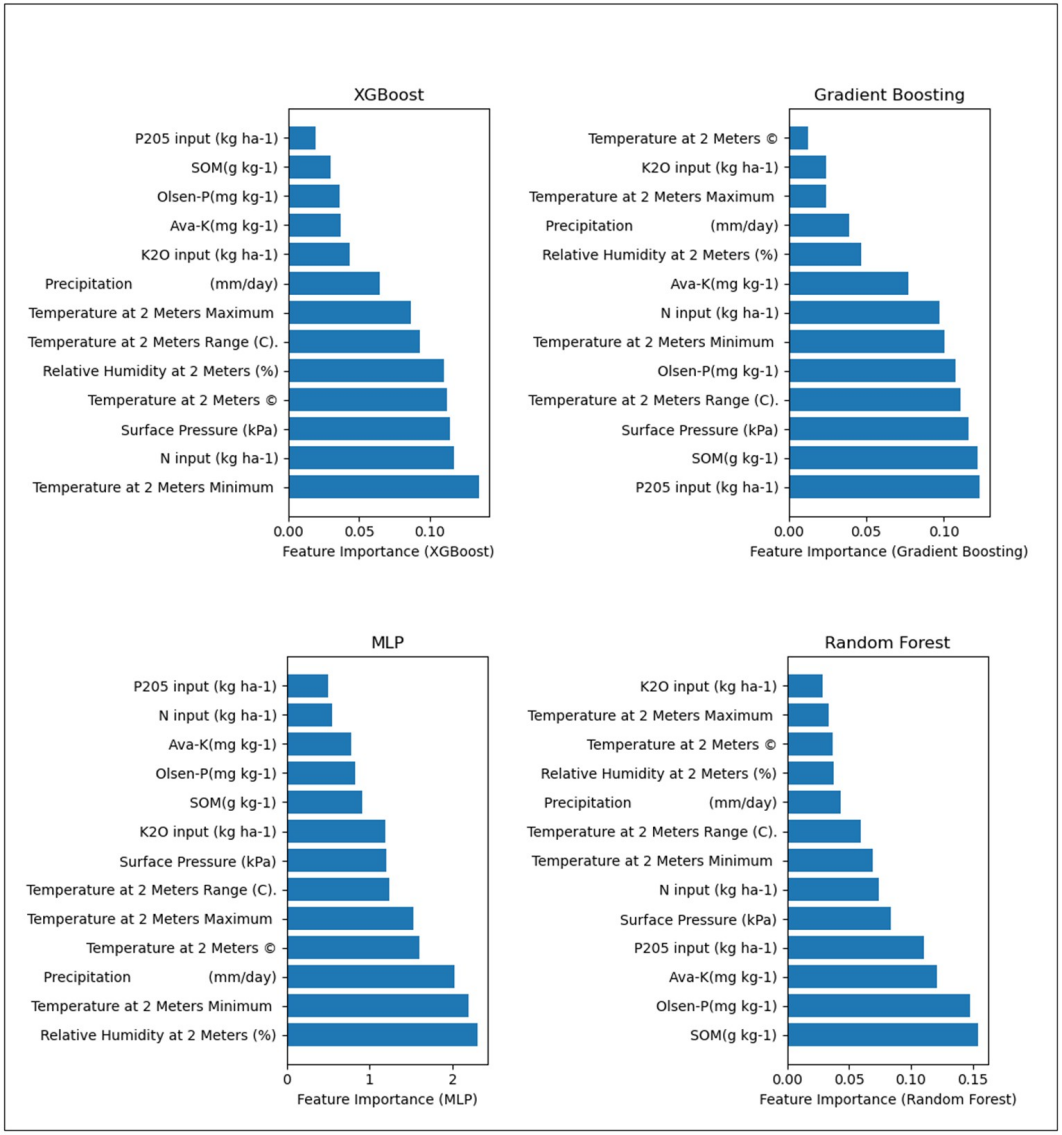
Comparison between feature importance of each model:XGB, GBR, MLP, and RF.

We studied the importance of the features to determine the weight of each feature using the best-performing algorithms: XGB, GBR, MLP, and RF. Feature importance scores were calculated based on how often a feature is used in constructing decision trees within the ensemble and how much those splits improve the model’s performance. For the MLP model, the importance of the features was determined using permutation importance. This method evaluates the impact of each feature by randomly permuting its values and measuring the resulting decrease in model performance, which is quantified by the change in mean squared error (MSE). Our exploration shows the significance of diverse features such as temperature at 2 meters altitude (°C), maximum temperature at 2 meters altitude, minimum temperature at 2 meters altitude, relative humidity at 2 meters altitude, precipitation (mm/day), and surface pressure (kPa) in relation to the yield of the maize cultivar Zhengdan 958 as shown in Fig 6.

XGB’s assessment of feature importance revealed that temperature at 2 meters minimum held the greatest influence over the yield response of the ZhengDan 958 variety. Other key features included N input, surface pressure, temperature at 2 meters, and relative humidity at 2 meters, which all had a significant impact on the yield predictions. This highlights the importance of weather-related factors in XGB’s predictive model, particularly temperature. For Random Forest (RF), SOM (Soil Organic Matter) emerged as the most dominant feature influencing yield prediction, followed by Olsen-P, Ava-K, and P205 input. RF’s reliance on soil attributes underscores its focus on nutrient availability and soil health as major determinants of crop yield, with weather features playing a comparatively lesser role. In the case of Gradient Boosting Regressor (GBR), P205 input was identified as the most important feature, followed by SOM, surface pressure, and temperature at 2 meters range. This suggests that while soil properties like P205 and SOM play a significant role, GBR also places substantial emphasis on weather-related factors, balancing soil and weather influences more evenly than RF. The MLP model, on the other hand, ranked relative humidity at 2 meters as the most important feature, with temperature at 2 meters minimum, precipitation, and temperature at 2 meters maximum following closely. This indicates that MLP’s predictive capability is primarily driven by atmospheric conditions, particularly humidity and temperature. These findings emphasize the critical role that temperature-related features play across all models, particularly in the case of XGB and MLP. Additionally, soil properties like P205, SOM, and Olsen-P were consistently important for models like RF and GBR, demonstrating the varied importance that each model assigns to weather and soil factors. Notably, XGB and GBR showed greater agreement in prioritizing both soil and weather features, while RF placed more emphasis on soil attributes, and MLP leaned more towards atmospheric conditions.

The outcomes of feature importance underscore a consistent preference for weather traits (as showcased in Fig 6, alongside the importance of nutrient availability and nutrient status attributes. In addition, we conducted a stepwise regression analysis. The key variables identified included P205 input (kg ha-1)’, ‘K2O input (kg ha-1)’, ‘Olsen-P(mg kg-1)’, ‘Ava-K(mg kg-1)’, ‘SOM(g kg-1)’, ‘Surface Pressure (kPa)’, ‘N input (kg ha-1)’, which were selected based on their statistical significance. A one-unit increase in these parameters is associated with a significant increase in yield (in kg/ha), while holding all other variables constant. Evidently, the inclusion of soil and weather features is likely to improve the predictive precision of maize yield for the Zhengdan 958 hybrid. The full test results can be found in supplementary Material (S3 Table).

## Discussion

Using a large collection of maize observations from China, we undertook a comprehensive analysis to summarize the changes in yield response across varying fertilizer rates, soil attributes, and weather conditions. Our investigation hinged on the utilization of the summer maize cropping system and the cultivar ZhengDan 958 as our focal study crop. An important step of our approach involved the integration of weather data, particularly historical and site-specific information, signifying a crucial stride towards enhancing the robustness and precision of yield predictions. Within this study, we directed our attention towards assessing the efficacy of two emerging gradient-boosting machine learning algorithms, namely XGB and GBR, in the prediction of ZhengDan 958’s yield. This endeavor marks the first time this particular cultivar has been used as the benchmark for comparing diverse machine-learning algorithms. To facilitate this comparative analysis, we compiled a multi-source dataset to study the most powerful algorithm and key features for corn yield prediction. Our study has underscored the feasibility of harnessing machine learning techniques to generate site-specific yield prediction. While prior research has acknowledged the efficacy of random forest models in predicting corn production [1, 33, 34], our investigation reveals that GBR and XGB surpass the performance of the random forest algorithm using the case of the ZhengDan 958 data. A recent comparative analysis conducted by [13] also highlighted the outperformance of XGBOOST compared to alternative algorithms. Moreover, a review by [35] that evaluated over fifty studies in crop yield prediction underscored the prevalence of gradient-boosting trees and random forests as the preferred algorithms within this domain, in concordance with our findings. Similarly, a study by Burdett et al. employed multiple linear regression, artificial neural networks, decision trees, and random forests to establish methodologies capable of associating soil attributes with crop yields at a subfield scale. The features included topographic attributes and soil nutrient data such as pH, soil organic matter content (OM), cation exchange capacity (CEC), soil phosphorus content, zinc (Zn), potassium (K), elevation, and topographic moisture index. Among these techniques, random forests exhibited superior performance, achieving an *R*^2^ value of 0.85 for corn yield prediction [34]. Furthermore, our integrated dataset analysis underscores GBR and XGBOOST as contenders for robust yield prediction and significant attribute selection, as compared to RF and MLP. In addition to the predictive power attributed to novel algorithmic enhancements, the performance observed is also influenced by the array of environmental and soil properties [13, 36]. Prior research endeavors have often focused on factors such as soil characteristics, fertilizer utilization, and management methodologies to predict crop yields, often neglecting the substantial impact of weather and environmental attributes [29]. However, these neglected elements are worthy of attention as determinants of crop growth. Recent trends have witnessed an increasing integration of weather features into analyses, facilitated by the availability of expansive datasets offering real-time or historical weather information through APIs. Feature selection outcomes have underscored the influence of independent attributes, highlighting the efficacy of weather features in yield prediction [37]. Among them, precipitation, temperature, and surface pressure have been consistently favored by top-performing algorithms.

**Fig 7:**
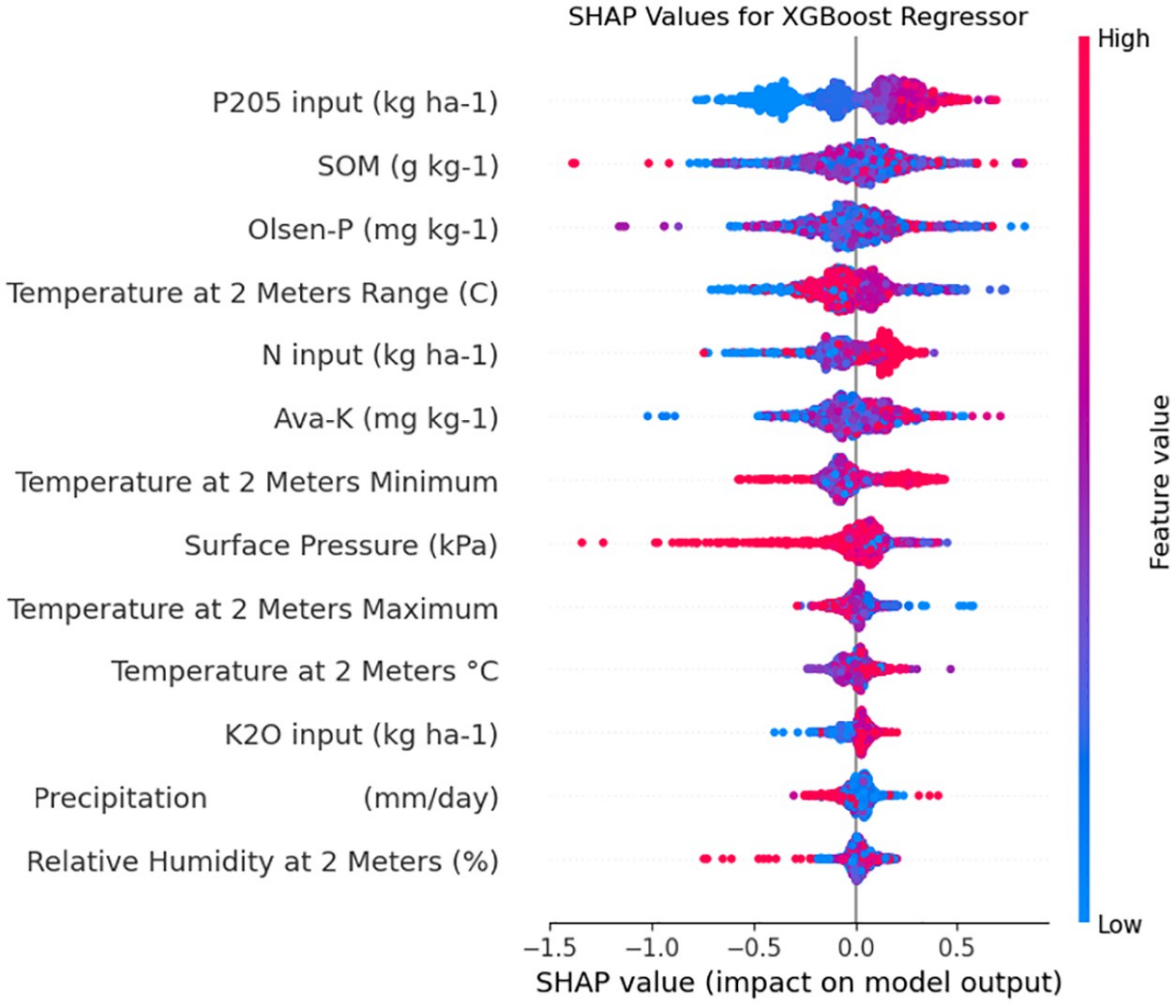
SHAP values of feature importance of the best performing model XGB.

For instance, the inclusion of these features resulted in a significant enhancement in GBR’s performance, reflected in a reduction of RMSE from 0.76 to 0.39, and an improvement in *R*^2^ and MAE from 0.43, 0.58 to 0.86 and 0.27, respectively. For XGBoost, the optimized model achieved an RMSE of 0.37, *R*^2^ of 0.87, and MAE of 0.26, which represents an improvement from 0.47, 0.78, and 0.34, respectively. MLP also showed moderate performance with an RMSE of 0.51, an *R*^2^ of 0.75, and an MAE of 0.38, which represents an improvement from 0.68, 0.54, and 0.51, respectively. In contrast, RF yielded the lowest performance, with an RMSE of 0.71, *R*^2^ of 0.51, and an MAE of 0.56, which represents a deterioration from 0.65, 0.58, and 0.48, respectively. The results of the models without the weather feature are presented in the Supplementary Material (S1 Table). These results clearly indicate the superiority of gradient-boosting methods like XGB and GBR over RF and MLP in predicting maize hybrid yields. The outcomes also underscore the notable role of soil attributes, particularly SOM, NPK availability, and utilization, in yield prediction. Furthermore, the integration of diverse data sources has been found to improve the model’s accuracy and robustness. Hence, instead of relying solely on a singular dataset, a holistic approach including various data sources is advocated [1, 13, 38] Feature contributions can also be measured via SHapley Additive exPlanations (SHAP) values. Features that better contribute to the prediction are associated with higher SHAP values. Fig 7 shows the SHAP values of the features used in our study when using GBR for prediction. For example, the figure shows that several observations (red points positioned in the left of x-axis) are negatively correlated with ‘Surface Pressure’. The figure also shows the importance of ‘P205 input’ along with the two weather features as the top three contributing features.

## Conclusions

In summary, this study demonstrates the successful application of machine learning algorithms to produce accurate and timely yield estimates for the maize variety Zhengdan 958 in China. By integrating various data sources, including soil features and weather characteristics, we developed machine learning models that provide accurate, site-specific yield predictions. Among the models tested, XGBoost proved to be the best performing, closely followed by GBR, underlining the superiority of gradient boosting techniques in yield prediction. Although the MLP model also delivered reasonable results, it lagged behind XGBoost and GBR, further underlining the dominance of these gradient boosting algorithms. The inclusion of weather features, particularly temperature and humidity, significantly improved the prediction accuracy of both XGBoost and GBR, as evidenced by their strong performance metrics. This result underscores the crucial role of environmental factors in crop yield modeling, alongside traditional soil attributes such as P205, SOM and Olsen-P. Our results support the growing realization that machine learning, when properly applied, has the potential to transform agricultural practices by improving yield predictions and supporting data-driven decisions. This study also suggests that future research should incorporate long-term field trials to further refine yield prediction models, especially for specific varieties such as Zhengdan 958. These efforts can contribute to the development of personalized decision support systems tailored to smallholder farmers, enabling more accurate yield predictions, higher nutrient efficiency and better economic returns. Such systems can play an important role in optimizing fertilizer use, reducing environmental impacts and ultimately increasing crop productivity. However, further research is needed to develop even more robust models that incorporate additional variables such as environmental impacts and residual nutrient inputs to the soil. Creating reliable indices to measure these multiple influences will be critical to improving model accuracy and applicability. Overall, this study sets the stage for a broader application of machine learning techniques in agriculture and highlights their potential to make informed decisions and promote sustainable agricultural practices.

## Supporting information

S1 Fig Frequency distribution of the most selected features used in the current study. The histogram visualizes the importance of various features.
(TIF)

S2 Fig Residual plots for model evaluation. These plots help assess the accuracy and errors in predictions.
(TIF)

S3 Fig Residual histograms for models. This figure illustrates the distribution of residuals, showcasing the deviations.
(TIF)

S1 Table Model performance comparison. Detailed performance metrics (R², MSE, RMSE, and MAE) for each model evaluated in the study.
(PDF)

S2 Table Descriptive statistics for features. Summary statistics (Mean, Std, Min, Max) for all input variables used in the models.
(PDF)

S3 Table Stepwise selected features. Features selected for the models based on their statistical significance (p-values).
(PDF)

S4 Table Predictive performance testing and training results with CV errors. Metrics for training and testing datasets, and cross-validation errors across models.
(PDF)

S5 Table Diebold-Mariano test results. Comparative statistics for model accuracy between pairs of models.
(PDF)
