# Supplementary figures and images for: Gradient boosting for yield prediction of elite maize hybrid ZhengDan 958
Source: PLoS One. 2024 Dec 17;19(12):e0315493. doi: 10.1371/journal.pone.0315493 (PMC11651618; doi:10.1371/journal.pone.0315493)

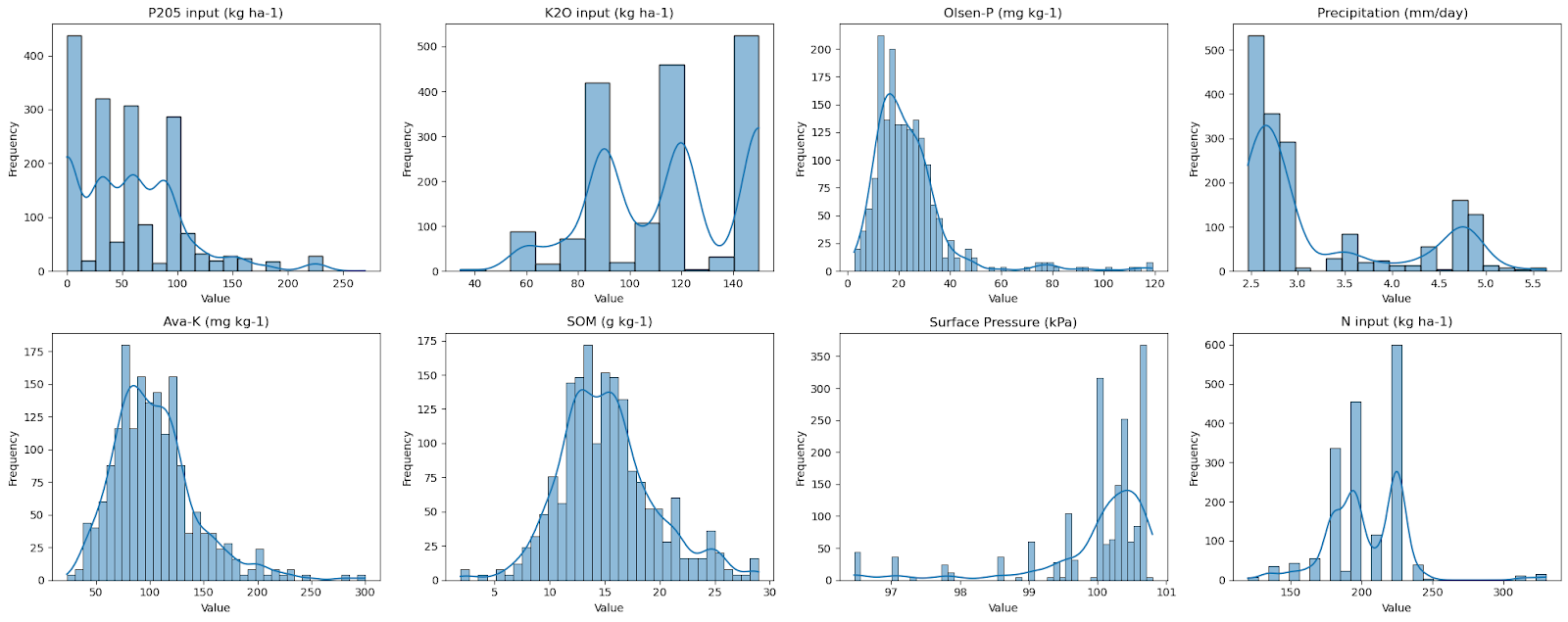

Supplement: S1 Fig — The histogram visualizes the importance of various features. (TIF) [file pone.0315493.s001.tif]

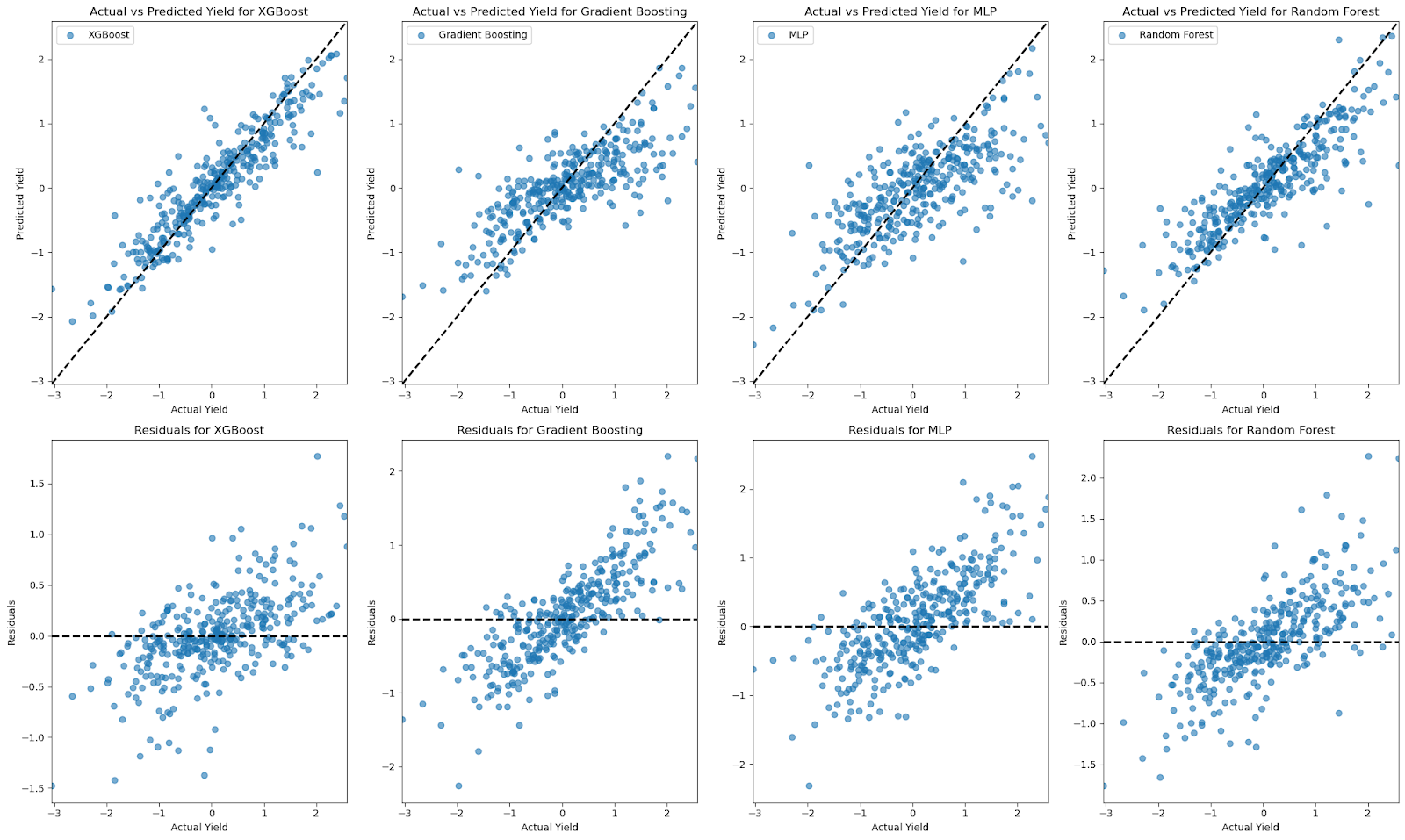

Supplement: S2 Fig — These plots help assess the accuracy and errors in predictions. (TIF) [file pone.0315493.s002.tif]

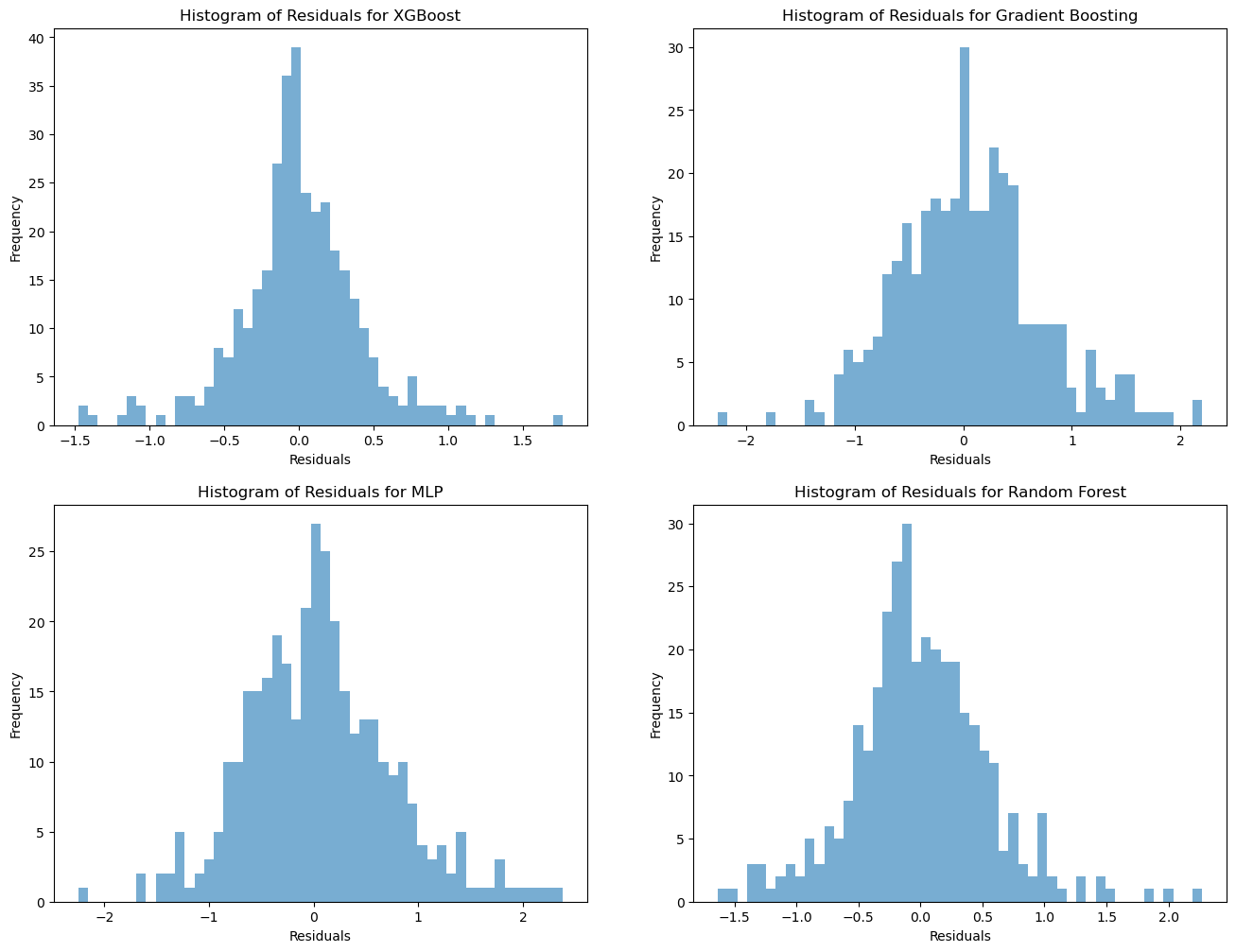

Supplement: S3 Fig — This figure illustrates the distribution of residuals, showcasing the deviations. (TIF) [file pone.0315493.s003.tif]
