# Supplementary material for: Gradient boosting for yield prediction of elite maize hybrid ZhengDan 958
Source: PLoS One. 2024 Dec 17;19(12):e0315493. doi: 10.1371/journal.pone.0315493 (PMC11651618; doi:10.1371/journal.pone.0315493)
Supplement: S1 Table — Detailed performance metrics (R², MSE, RMSE, and MAE) for each model evaluated in the study. (PDF) [file pone.0315493.s004.pdf]

Table S1: Model Performance Comparison

Model	R2-test	MSE-testdata	RMSE	MAE
XGBRegressor	0.782	0.224	0.474	0.342
RandomForestRegressor	0.589	0.423	0.650	0.481
MLPRegressor	0.549	0.464	0.681	0.514
GradientBoostingRegressor	0.437	0.580	0.761	0.583
KNeighborsRegressor	0.330	0.689	0.830	0.629
DecisionTreeRegressor	0.299	0.722	0.850	0.571
SVR	0.227	0.898	0.796	0.670
LinearRegression	0.127	0.948	0.741	0.670
