# Supplementary material for: Gradient boosting for yield prediction of elite maize hybrid ZhengDan 958
Source: PLoS One. 2024 Dec 17;19(12):e0315493. doi: 10.1371/journal.pone.0315493 (PMC11651618; doi:10.1371/journal.pone.0315493)
Supplement: S2 Table — Summary statistics (Mean, Std, Min, Max) for all input variables used in the models. (PDF) [file pone.0315493.s005.pdf]

Table S2: Descriptive Statistics for the Features

	Mean	Std	Min	Max
P205 input (kg ha^{-1})	55.24	48.23	0.00	270.00
K2O input (kg ha^{-1})	115.30	28.23	34.50	150.00
Olsen-P (mg kg^{-1})	23.90	15.98	2.60	119.00
Ava-K (mg kg^{-1})	104.27	39.18	23.00	300.00
SOM (g kg^{-1})	15.23	4.41	1.90	29.00
Surface Pressure (kPa)	99.98	0.92	96.46	100.80
N input (kg ha^{-1})	202.89	28.31	120.00	330.00
Temperature at 2 Meters Range (C)	12.51	0.95	4.78	13.47
Precipitation (mm/day)	3.22	0.87	2.47	5.63
