# Supplementary material for: Gradient boosting for yield prediction of elite maize hybrid ZhengDan 958
Source: PLoS One. 2024 Dec 17;19(12):e0315493. doi: 10.1371/journal.pone.0315493 (PMC11651618; doi:10.1371/journal.pone.0315493)
Supplement: S3 Table — Features selected for the models based on their statistical significance (p-values). (PDF) [file pone.0315493.s006.pdf]

Table S3: Stepwise Selected Features

**Feature**	**p-value**
P205 input (kg ha^{-1})	$1.2809728365166646 \times 10^{-21}$
K2O input (kg ha^{-1})	$5.206508881837194 \times 10^{-18}$
Olsen-P (mg kg^{-1})	$3.319133551279681 \times 10^{-8}$
Ava-K (mg kg^{-1})	0.00023940471010240553
SOM (g kg^{-1})	0.0005526950884943553
Surface Pressure (kPa)	0.009075400218386119
N input (kg ha^{-1})	0.009007153039060469
