# Supplementary material for: Gradient boosting for yield prediction of elite maize hybrid ZhengDan 958
Source: PLoS One. 2024 Dec 17;19(12):e0315493. doi: 10.1371/journal.pone.0315493 (PMC11651618; doi:10.1371/journal.pone.0315493)
Supplement: S4 Table — Metrics for training and testing datasets, and cross-validation errors across models. (PDF) [file pone.0315493.s007.pdf]

Table S4: The predictive performance of different models

Model	R2-TR	R2-TS	RMSE-TR	RMSE-TS	MAE-TR	MAE-TS	MSE-CV
XGB	0.99	0.84	0.11	0.41	0.07	0.29	0.22
MLP	0.90	0.74	0.31	0.52	0.22	0.38	0.35
RF	0.95	0.71	0.22	0.55	0.15	0.40	0.35
KNN	0.70	0.59	0.54	0.65	0.40	0.49	0.55
GBR	0.65	0.57	0.59	0.66	0.44	0.51	0.45
DTR	0.99	0.47	0.07	0.74	0.01	0.47	0.64
SVR	0.47	0.43	0.72	0.77	0.48	0.56	0.64
LR	0.14	0.13	0.93	0.95	0.72	0.74	0.87
