# Supplementary material for: Gradient boosting for yield prediction of elite maize hybrid ZhengDan 958
Source: PLoS One. 2024 Dec 17;19(12):e0315493. doi: 10.1371/journal.pone.0315493 (PMC11651618; doi:10.1371/journal.pone.0315493)
Supplement: S5 Table — Comparative statistics for model accuracy between pairs of models. (PDF) [file pone.0315493.s008.pdf]

Table S5: Diebold-Mariano Test Results

**Model 1**	**Model 2**	**Statistic**
XGBoost	Gradient Boosting	8.4819
XGBoost	MLP	8.0641
XGBoost	Random Forest	8.6947
Gradient Boosting	MLP	8.3930
Gradient Boosting	Random Forest	9.1299
MLP	Random Forest	7.9092
